# Dr. Hennen

**Published:** 1829-02

**Authors:** 


Died lately, at Gibraltar, Dr. Hennen, Inspector of Army Hospitals. From the first dawning of his professional career to the last hour of his existence, he gave very many proofs of the ardent and honourable zeal with which he cultivated every pursuit that could be usefully employed in the different responsible situations in which he was placed. Dr. Hennen received from the Emperor of Russia a valuable diamond ring, as a testimony of his Majesty's gratitude for the important services he had conferred upon the armies of modern Europe. We understand Dr. H. was lately occupied in preparing for publication a new edition of his work on Military Surgery.

